# Detection of brain regions responsible for chronic pain in osteoarthritis: an fMRI-based neuroimaging study using deep learning

**DOI:** 10.3389/fneur.2023.1195923

**Published:** 2023-06-02

**Authors:** Indranath Chatterjee, Lea Baumgartner, Migyung Cho

**Affiliations:** ^1^Department of Computer Engineering, Tongmyong University, Busan, Republic of Korea; ^2^School of Technology, Woxsen University, Telangana, India; ^3^Department of Media, Hochschule der Medien, University of Applied Science, Stuttgart, Germany; ^4^Department of Game Engineering, Tongmyong University, Busan, Republic of Korea

**Keywords:** functional magnetic resonance imaging (fMRI), medical imaging, osteoarthritis, chronic pain, deep learning, classification

## Abstract

**Introduction:**

Chronic pain is a multifaceted condition that has yet to be fully comprehended. It is frequently linked with a range of disorders, particularly osteoarthritis (OA), which arises from the progressive deterioration of the protective cartilage that cushions the bone endings over time.

**Methods:**

In this paper, we examine the impact of chronic pain on the brain using advanced deep learning (DL) algorithms that leverage resting-state functional magnetic resonance imaging (fMRI) data from both OA pain patients and healthy controls. Our study encompasses fMRI data from 51 pain patients and 20 healthy subjects. To differentiate chronic pain-affected OA patients from healthy controls, we introduce a DL-based computer-aided diagnosis framework that incorporates Multi-Layer Perceptron and Convolutional Neural Networks (CNN), separately.

**Results:**

Among the examined algorithms, we discovered that CNN outperformed the others and achieved a notable accuracy rate of nearly 85%. In addition, our investigation scrutinized the brain regions affected by chronic pain and successfully identified several regions that have not been mentioned in previous literature, including the occipital lobe, the superior frontal gyrus, the cuneus, the middle occipital gyrus, and the culmen.

**Discussion:**

This pioneering study explores the applicability of DL algorithms in pinpointing the differentiating brain regions in OA patients who experience chronic pain. The outcomes of our research could make a significant contribution to medical research on OA pain patients and facilitate fMRI-based pain recognition, ultimately leading to enhanced clinical intervention for chronic pain patients.

## 1. Introduction

Chronic pain is a complicated phenomenon in the medical field, and its root cause remains poorly understood. It is characterized by constant pain that surpasses the typical healing time and lasts for at least three to 6 months. This type of pain occurs in various diseases, with arthritis being one of the most prominent. Arthritis is a broad term that refers to inflammation of the joints, which can occur in acute or chronic forms. Inflammation is typically a response to irritants such as bacteria or foreign objects and leads to swelling and discomfort and can eventually result in chronic diseases. Among the various types of arthritis, Osteoarthritis (OA) is the most common. Normally, cartilage serves to protect joints between bones, but in OA, cartilage degenerates, causing bones to rub against each other, resulting in pain, stiffness, and reduced mobility. OA affects an estimated 20% of adults in the United States, and it is a leading cause of disability and lost productivity. According to data from the National Health Interview Survey (NHIS), OA affects over 32.5 million adults in the USA alone, with the projected number of patients to rise to nearly 67 million by 2030 (1, 2). For several reasons, studying and conducting research on OA is decisive. OA is a common and debilitating disease that affects a large number of people, particularly older adults. It can also decrease the quality of life, imposing a significant economic and social burden on individuals and society. According to the Centers for Disease Control and Prevention, the total annual costs of OA in the United States alone in 2015 were estimated to be $65 billion (3).

While the specific root cause of OA is unknown, researchers have identified several modifiable risk factors, such as obesity, joint injury, and physical inactivity, that can be used to inform prevention and treatment strategies (4). Additionally, studies (5, 6) on the neural mechanisms underlying pain in OA patients can help us understand the disease and develop more effective treatments. Researchers may be able to develop more targeted and effective treatments for chronic pain by identifying specific brain regions and networks involved in pain processing in OA patients, including non-pharmacological interventions such as cognitive-behavioral therapy and novel pharmacological approaches (7) targeting the central nervous system. In addition, studies into the neural mechanisms underlying chronic pain in OA may have broader implications for other chronic pain conditions like fibromyalgia, chronic back pain, and neuropathic pain, which share some characteristics with OA. As a result, studying OA has the potential to not only improve the lives of people with OA but also to advance our understanding of chronic pain in general. The high prevalence and significant impact of OA on public health highlight the critical need for ongoing research efforts aimed at advancing our understanding of the disease’s underlying mechanisms and developing effective diagnostic and treatment strategies. Although current treatments for chronic pain and OA are available, they can be ineffective or associated with side effects, such as addiction or dependency on pain medication, making it difficult to manage these conditions effectively (8).

Incorporating neuroimaging studies to understand the neurobiological basis of chronic pain can lead to the development of more targeted and effective pain management strategies. By identifying the brain regions involved in chronic pain and developing deep learning (DL) frameworks for pain diagnosis and treatment, this study has significant implications for improving the quality of life of individuals with chronic pain and OA. Functional magnetic resonance imaging (fMRI) is one of the preferred neuroimaging tools for capturing brain activity related to pain sensation (9). Resting-state (rs) fMRI is a technique used to measure intrinsic brain activity in the absence of any specific task or stimulus, by detecting the low-frequency fluctuations in BOLD signals that reflect functional connectivity between different brain regions (10). The fMRI identifies the brain’s functional activity by measuring blood oxygenation level-dependent changes (BOLD) using the hemodynamic response function. Regions of the brain that exhibit stronger functional connectivity show stronger correlations in the BOLD signal fluctuations detected by rs-fMRI, whereas regions with weaker functional connectivity exhibit weaker correlations. In rs-fMRI, functional connectivity is measured by assessing the temporal correlation of BOLD signal fluctuations between different brain regions, without the need for external stimulation (11).

Several ML and DL techniques have been successfully used in brain research studies using fMRI data. Despite breakthroughs in the use of rs-fMRI and ML and DL architectures in brain research, little work has been done to examine chronic pain using these approaches. This study aims to contribute to the understanding of the neurobiological basis of chronic pain and OA by analyzing rs-fMRI data of chronic pain patients. Specifically, the hypothesis for this study is that DL frameworks can be developed to analyze rs-fMRI data of chronic pain patients and identify potential brain regions responsible for pain sensation in OA. Despite contemporary treatments for chronic pain and osteoarthritis, effective management remains difficult, with pain medicines frequently causing side effects and addiction (12). Therefore, by identifying potential brain regions responsible for pain sensation, the study aims to develop a computer-aided diagnosis tool that could potentially lead to more effective management of chronic pain and OA.

To achieve these objectives, the study proposes a novel two-stage classification approach that consists of a general linear model and parameter-optimized neural network-based algorithms. This approach is unique and innovative, as it employs DL architectures in the domain of chronic pain study of OA patients using rs-fMRI data for the first time. The proposed approach also evaluates the brain activity pattern in chronic pain by identifying probable brain areas that trigger pain feeling. The successful development of such a tool would have significant implications for improving the quality of life of individuals with chronic pain and OA by enabling more targeted and effective pain management strategies.

## 2. Investigating chronic pain in OA patients: insights from fMRI and ML studies

In recent years, there has been a growing interest in utilizing fMRI to investigate brain activity changes in individuals with chronic pain conditions, particularly those with OA. Several research studies have examined brain regions associated with pain within OA. For instance, Baliki et al. (13) conducted an fMRI study on analgesic treatment in chronic back pain and knee OA, which reported several brain regions associated with pain within OA, such as the thalamus, secondary somatosensory, insular, and cingulate cortices, with unilateral activity in the putamen and amygdala. Similarly, Sofat et al. (14) found that OA patients who experience pain may have particular brain activation components to explain their symptoms. Gwilym et al. (15) used fMRI to analyze pain perception in OA patients and compared the results to healthy controls. The study revealed that patients with OA had a lower threshold perception for feeling pain and displayed a higher activation in the brainstem.

While these fMRI studies have provided insight into the brain regions associated with pain perception in OA patients, there is a need to explore the potential of ML and DL algorithms in discovering additional brain regions involved in chronic pain suffering. Although previous research has employed DL architectures on OA data, the focus was not on pain as a symptom but rather on OA in general. For instance, Tiulpin et al. (16) used a Deep Siamese convolutional neural network (CNN) to score knee OA severity automatically using plain radiographs, while Xue et al. (17) trained a deep CNN to diagnose hip OA automatically from X-ray images.

On the other hand, several studies have investigated the application of DL algorithms on neuroimaging data for analyzing state-of-the-art methodologies. Wang et al. (18) explored 3D CNNs with embedded dilated convolutional layers for 3D brain MRI data classification, while El Gazzar et al. (19) used 1D-CNNs for rs-fMRI data to classify autism using the ABIDE I + II dataset. Although most of the studies applied DL algorithms to various data-capturing cognitive impairment problems, such as ADHD or autism, only a few studies investigated the brain activity caused by pain based on fMRI data.

Santana et al. (20) used rs-fMRI data using ML and DL methods to study the functional connectivity patterns in the brains of chronic pain patients and healthy controls. The study included 98 healthy volunteers and 60 chronic pain patients, including those with fibromyalgia and back pain. Santana et al. calculated functional connectivity inside the brains using time series from fMRI data and used correlation and dynamic time warping distance (DTW) to evaluate these connectivity values. Following the computation of the connection matrix, the authors utilized z-score normalization and four distinct classifiers, notably BrainNetCNN and TPOT4, on the data. The study demonstrated that rs-fMRI data can be used to find a potential biomarker of chronic pain conditions, which could aid in better understanding the neural mechanisms underlying chronic pain in osteoarthritis patients.

Recent studies have shown that imagery-based interventions hold promise for managing chronic pain in various populations. The potential of mental and motor imagery (MI) in managing central neuropathic pain (CNP), complex regional pain syndrome type 1 (CRPS-1), and central neuropathic pain in people with spinal cord injury (SCI) have been demonstrated by Kaur et al. (21), another study (22), and a third study (23), respectively. These findings suggest that imagery-based interventions could be effective for managing chronic pain in diverse populations, including OA patients.

Although fMRI has been utilized to identify brain regions associated with pain perception in OA patients, there is a need to explore the potential of ML and DL algorithms to discover additional brain regions involved in chronic pain suffering. The application of DL algorithms on neuroimaging data for analyzing state-of-the-art methodologies has shown promising results. While only a few comparable studies have been found, more research is needed to investigate the classification of pain patients using fMRI data based on DL algorithms. In this work, we propose that ML and DL algorithms could play a significant role in better understanding the neural mechanisms underlying chronic pain in osteoarthritis patients.

## 3. Dataset

### 3.1. Dataset details

The fMRI data for this study on OA patients with chronic pain were obtained from OpenFMRI (24) and have been assigned the accession number ds000208. This dataset comprises resting-state BOLD fMRI data from 76 subjects, including 20 healthy controls and 56 OA chronic pain patients. The data was initially collected for Tetreault et al.’s (24) study, and Table 1 provides an overview of the subjects’ demographics. The patients were divided into three different groups to analyze the effect of pain treatment with drugs compared to placebo. However, the fMRI data used in this study was acquired before the treatment and may have two groups instead of four, namely healthy controls and OA pain patients. Table 2 provides an overview of the patients after treatment.

**Table 1:** Demographic details of the subjects within the study of Tetreault et al. (24).

Participants	No. of subjects	Male/female	Age (Mean ± Std Dev)
Healthy control	20	10/10	57.9 ± 6.66
2W-Placebo patient	17	8/9	56.88 ± 5.68
3M-Placebo patients	20	9/11	57.6 ± 9.51
3M-Duloxetine patients	19	9/10	59.16 ± 4.61

**Table 2:** Chosen data for a balanced dataset for the second experiment training ML and DL algorithms.

Participants	No. of subjects	Male/female	Age (Mean ± Std Dev)
Healthy control	20	10/10	57.9 ± 6.66
Patients	51	25/26	57.8 ± 6.98

### 3.2. Imaging parameters

The fMRI data were obtained using a 3T Siemens Trio whole-body scanner with echo-planar imaging (EPI) capability and were delivered as 4-D NIFTI files. The data consists of voxels with dimensions of 3.438 × 3.438 × 3 millimeters and 300 volumes. The other imaging parameters include a repetition time (TR) of 2.5 s, echo time (TE) of 30 ms, flip angle of 90 degrees, number of slices of 40, slice thickness of 3 mm, and in-plane resolution of 64 × 64.

### 3.3. Preprocessing

To fully utilize the potential of the brain images in the dataset, state-of-the-art preprocessing techniques were applied using the CONN toolbox (25). This pipeline consists of several steps, including functional realignment and unwarp, slice-timing, outlier identification, direct segmentation, normalization, and smoothing (26), which were implemented to ensure high-quality data.

Firstly, the CONN toolbox uses SPM’s realign and unwarp functions to co-register and resample all scans of one subject to a reference image. This reference image is the first scan of the session, ensuring that the samples are well-aligned concerning distortion-by-motion artifacts, such as head movements. Following this, slice-timing correction is applied for temporal correction (25).

The outlier identification step identifies outlier scans by estimating the global BOLD signal. The framewise displacement is calculated by applying a bounding box around the brain with the size of 140 × 180 × 115 mm, and the global BOLD signal change is calculated by computing the average change at each time point. This results in a list of potential outliers, a list of global BOLD change and head-motion measures, and a new reference image, excluding the outlier scans.

During the direct segmentation and normalization step, the data is normalized into a standard MNI (Montreal Neurological Institute) space. This procedure uses the mean BOLD signal reference image for segmentation and normalization, resulting in resampled data with a bounding box size of 180 × 216 × 180 mm, with [2 × 2 × 2] isotropic voxels (25).

Lastly, the data undergoes functional smoothing, where spatial convolution with a Gaussian kernel of 8 mm full-width half maximum (FWHM) is applied to reduce the influence of inter-subject variations in anatomy and to increase the BOLD signal-to-noise ratio. The final output yields an image size of (91 × 109 × 91), ensuring a high-quality dataset for further analysis (25).

### 3.4. First-level analysis (GLM)

Upon completion of the preprocessing steps, statistical analysis is performed using the Statistical Parametric Mapping (SPM12) toolbox, which employs correlation analysis or advanced modeling methods to identify voxels with high activation (27). The result of this analysis is a statistical map, commonly referred to as an activation map, that indicates the voxels with high activation during the scan.

The most widely used method for independently analyzing the voxel’s time series is the standard General Linear Model (GLM) analysis, which is a univariate analysis technique (11). Since this method fits a model to each voxel over time, the data for each voxel comprises a 1-D vector of intensity values, one for each time point (28). In the GLM analysis, the output data is denoted as y(t), which represents the predicted variable, while the model is represented by x(t). The slope or coefficient is denoted as β, and c is the intercept or constant, which represents the baseline intensity value. Lastly, e represents the error in the fitting of the model. The linear modeling can be expressed as y(t)=β×x(t)+c+e(t). The model fitting process involves adjusting the baseline and height of the square wave to fit the data, while the error term accounts for the residual error between the data and the fitted model.

The output data of this processing step is an activation map of brain areas over the volumes, which cannot be considered a standard image anymore. Therefore, the activation map provides information about the functional activity in various brain areas and is an essential tool for understanding brain function and identifying abnormalities (27).

### 3.5. Data preparation

Following preprocessing and GLM analysis, the data were manually inspected for cleanliness and accurate identification of brain areas. Five subjects had to be excluded from the total dataset due to the presence of excessive noise. Consequently, the preprocessed dataset includes 51 chronic pain patients and 20 healthy controls. A summary of the demographics of the dataset is presented in Table 2.

The inspection of the preprocessed data is crucial to ensure the quality of the dataset and eliminate any potential errors. Furthermore, the exclusion of subjects with excessive noise is necessary to ensure accurate and reliable analysis results. The resulting dataset comprises a sample size that is considered sufficient to produce valid and meaningful statistical inferences (29). The demographic information provided in Table 2 enables the reader to understand the characteristics of the study population and assess the generalizability of the findings.

The sample size in our study is uneven between the healthy control (HC) and osteoarthritis (OA) groups, with 20 HCs and 51 OA patients. The sample size, however, was determined by the availability of data that fulfilled our inclusion criteria, which needed matching imaging parameters and acquisition properties. Data that did not fulfill these criteria were eliminated, resulting in an unequal sample size. It is often challenging to obtain a perfectly balanced dataset in this type of research, given the limited availability of human subjects and the need to match subjects based on various demographic and clinical variables. Indeed, prior chronic pain studies have shown uneven sample sizes, with a greater number of patients than healthy controls (30, 31).

We used robust statistical procedures that can handle imbalanced data to address the issue of an unbalanced sample size. We used Welch’s *t*-test, which can handle varying variances and sample sizes (32). Permutation tests were also used, which produce accurate *p*-values even with unbalanced samples (33). We were able to obtain reliable statistical results and draw meaningful conclusions from our analysis thanks to these methods.

## 4. Experimental setup

**Figure 1:**
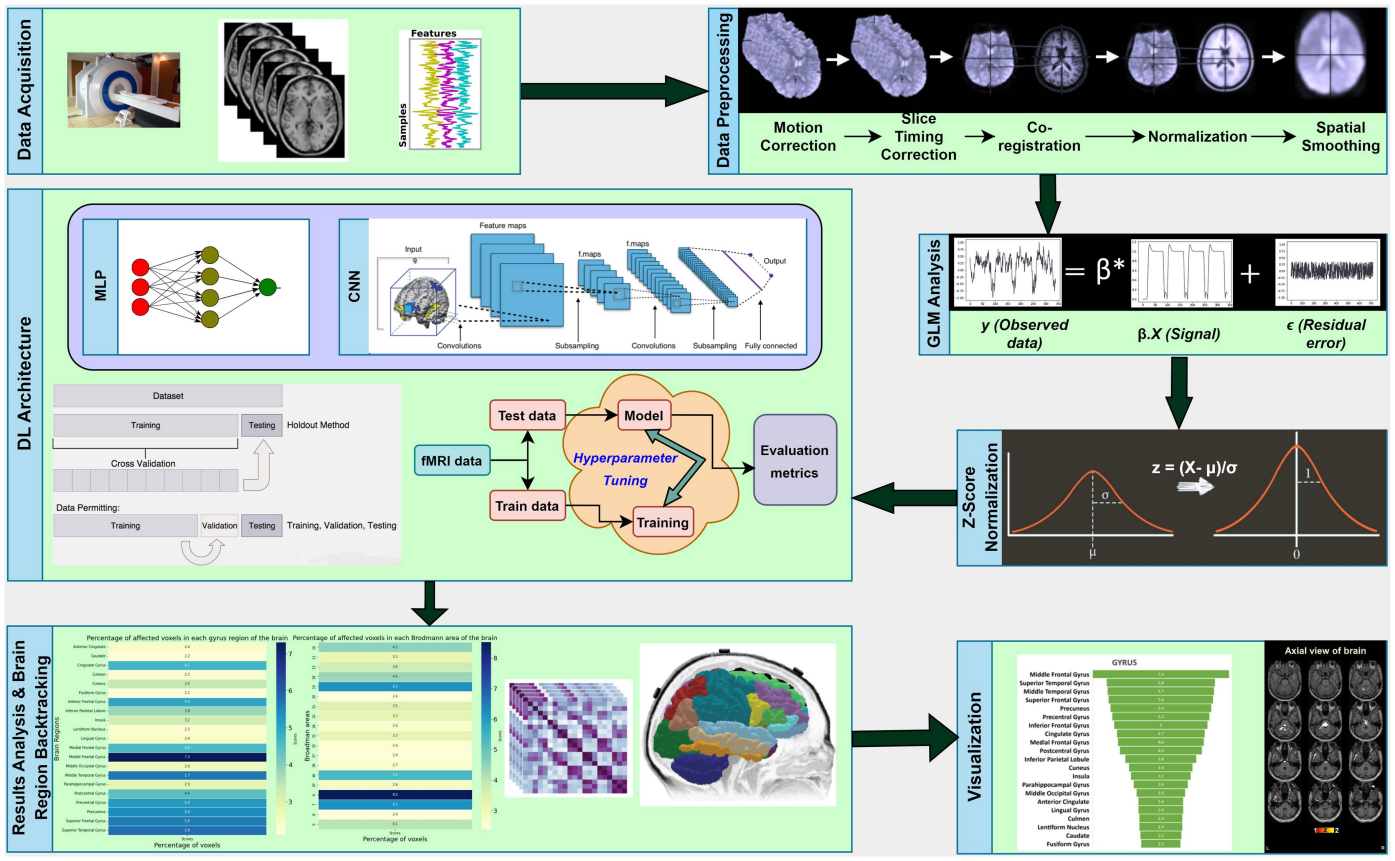
Overview of the workflow diagram showing the steps performed in this study.

In this section, we will describe the experimental setup used in our research, which involves the use of multi-layer perceptron (MLP) and CNN techniques. Figure 1 provides an overview of the working pipeline that we used in this study.

### 4.1. Theoretical background

#### 4.1.1. Multi-layer perceptron (MLP)

The MLP is a feed-forward neural network that can be used for classification and regression tasks. In contrast to recurrent networks, a feed-forward network has no feedback loops or internal state, and the network’s output is calculated based on the current input (34–36). The MLP is composed of an input layer, an output layer, and a specified number of hidden layers, each containing neural units modeled after human brain neurons. Each neuron in a layer receives input and generates output using an activation function that must be chosen based on the problem, the learning algorithm, and the network architecture.

In MLPs, the outputs produced by neurons in each layer are connected to all neurons in the subsequent layer, except for the last layer, which produces the final output. In this study, the problem at hand involves distinguishing between chronic pain patients and healthy controls, which is a binary classification problem.

#### 4.1.2. Convolutional neural network (CNN)

In this study, we have utilized CNN, which is a form of deep neural network architecture. CNN differs from ordinary neural networks, such as MLPs, in several ways (37). MLPs consist of fully connected layers where each neuron in one layer is connected to every unit in the next layer. This results in a large number of free parameters, and as the number of free parameters increases, so does the number of required training samples. To address this issue, CNNs limit the connections between neurons in one layer to a specific set of units in the next layer. This architecture enables filtered feature learning and allows certain neurons within the layers to be connected, depending on the domain.

The two primary components of CNNs are convolutional layers and max-pooling layers (38). Convolution is applied within the convolutional layers, which can be visualized using an image-processing example. The filter size (a;d), where a is the number of rows and d is the number of pixels per row in the filter, is an essential parameter for calculating the convolution. The input image I with r rows and e pixels per row is convolved by shifting the filter with size (a;d) for a particular feature f over all subregion positions within the input image I, where each subregion’s size equals the filter’s size (a;d). The number of possible positions is (r−a+1)·(e−d+1). The filter output at a subregion position calculates the scalar product between the filter’s coefficients and the values within the subregion.

The output of the hidden neurons is calculated with $h_{i}^{k}=g\left(W^{k}\cdot x_{i}+b^{k}\right)$, where $h_{i}^{k}\;$ is the ith output of the feature maps in layer k, and $x_{i}$ is the subregion of the input that constitutes the local receptive field of $h_{i}^{k}$. The filter coefficients of feature f within layer k are the weights $W^{k}$ between one neuron in the hidden layer and its local receptive field. Each feature $f_{k}$ in the hidden layer has a feature map containing as many hidden neurons as possible and shifts the filter across the input. All neurons in a feature map have the same weight matrix $W^{k}$, which results in CNNs having a low number of free parameters.

In the pooling layer, the region of A neurons in a feature map is partitioned into a small set of subregions, each consisting of B neurons. One value is calculated from the B neurons for each subregion during subsampling. There are three different types of subsampling methods: max-pooling, which computes the maximum of the subregion; min-pooling, which calculates the minimum; and mean-pooling, which calculates the mean value in the given subregion. CNNs can be trained using a gradient descent method, such as backpropagation. Although CNNs can be used for various problems, they are primarily used for image processing or classification.

### 4.2. Experimental setup

#### 4.2.1. Z-score normalization

Before proceeding with the training of the proposed DL algorithms, we undertook a critical pre-processing step using z-score normalization, commonly known as standardization. This normalization technique is widely used in ML to ensure that the data is on a standardized scale, which is crucial for the optimal performance of DL algorithms. Standardization aims to re-scale the values of the features such that they have a standard deviation of 1 and a mean of 0. We achieved this by applying the formula x=(value−μ)/σ, where μ represents the mean value of the features and σ represents the standard deviation across the feature. This crucial step enhances the efficiency of the training process by preventing features with larger values from having a dominant effect on the learning process.

For our study, we conducted experiments using preprocessed data from 51 patients with OA chronic pain and 20 healthy patients. The goal was to carry out a binary classification task, differentiating between healthy controls and pain patients. To achieve this, we implemented the code using Keras and TensorFlow. These are widely used and effective libraries in DL.

To optimize the training process and improve the accuracy of the binary classification task, we fine-tuned several configurations of hyperparameters. These included the number of layers, the number of filters within the layers, the learning rate, the optimizer, and the number of iterations. Hyperparameter optimization is a crucial step in training DL algorithms and ensures that the model performs optimally. We carefully selected the best configurations to achieve the highest possible accuracy in our binary classification task.

#### 4.2.2. MLP configurations

The parameter tuning of the MLP was performed using a comprehensive approach, where we experimented with different configurations of hidden layers and activation functions to achieve the best possible performance. We tested various numbers of hidden layers, including 3, 5, 7, and 10, and explored different activation functions, such as identity, logistic, and Rectified Linear Unit (ReLu) activation functions. Furthermore, we varied the batch sizes to 16, 32, and 64, and applied different optimization algorithms, such as Adam and Stochastic Gradient Descent (SGD). We also experimented with various learning rates, including 0.01, 0.001, and 0.0001.

Following rigorous experimentation, we obtained the most accurate configuration that yielded the best results so far. Specifically, we utilized five hidden layers, with the ReLu activation function applied within the layers. For training, we used a batch size of 32 and a learning rate of 0.0001. To optimize the training process, we used the SGD optimization algorithm, which is known to perform well in similar scenarios. Moreover, we conducted five cross-validation rounds, and each round consisted of 300 iterations. This approach was designed to systematically explore the parameter space of the MLP, ensuring that we identified the most effective configuration for our specific classification task.

#### 4.2.3. CNN configurations

The CNN training process was meticulously optimized by conducting a comprehensive search for the optimal values of various parameters. Specifically, the number of layers was varied and tested with values of 18, 32, and 51. Additionally, the filter size was fine-tuned using different configurations such as 128, 64, 32, and 16 filters applied within the architecture, with their respective value and max-pooling layers. Furthermore, a different number of dropout layers were experimented with. Various learning rates, ranging from 0.01 to 1e-7, were explored to identify the optimal rate for the training process. Different optimization algorithms such as SGD and Adam were also employed, and iterations ranging from 50 to 200 were tested.

Ultimately, the CNN architecture that exhibited the best performance was identified, consisting of five convolutional layers and two dense layers. The first three convolutional layers consisted of 16 filters, while the last two consisted of 32 filters. All convolutional layers were configured with a kernel size of 3 and the ReLu activation function. Max-pooling layers were added after each convolutional layer with a pool size of 2 and a stride of 1. Additionally, dropout layers were added after the third, fourth, and fifth max-pooling layers, with a dropout value of 0.5 in the first dropout layer and a value of 0.25 in the last two dropout layers. The first of the last two dense layers had 10 units with a ReLU activation function, and the last dense layer had one unit, providing the decision output with the Sigmoid activation function. The optimization algorithm chosen was SGD with a learning rate of 0.0000001. Finally, the training was performed with 150 epochs for each of the five rounds of cross-validation folds.

#### 4.2.4. Evaluation methods

All of the experiments in this study were evaluated using a 5-Fold Cross Validation (CV) technique and visualized using Receiver Operating Characteristic (ROC) Curve (34). When working with a relatively small dataset, the K-Fold CV technique can be used to improve the model evaluation by dividing the original dataset into $s_{l}$ partitions, where l is the index of one partition and one partition, $s_{l}$, is used as a validation or test set, while all other partitions are used as a training set. After training and testing on all possible combinations of training and test data, the mean accuracy of overall iterations can be calculated, leading to the selection of a combination with the lowest error rate.

In addition to the ROC curve, the Area Under the Curve (AUC) is also measured. The AUC value ranges from 0 to 1, and a model that predicts everything correctly has an AUC of 1.0. The advantage of using AUC is that it measures how well predictions are ranked, rather than the absolute values. It measures the quality of the model’s prediction regardless of the classification threshold.

In terms of model efficacy, we have also measured the model’s classification accuracy using the standard calculation of accuracy. However, since this study trained the neural networks on an imbalanced dataset, the numbers of true positives (TP), true negatives (TN), false positives (FP), and false negatives (FN) are important to evaluate the true performance of the algorithms, along with finding the Precision, Recall, and F1-score (34). These measures help to understand the performance of the model, especially when working with imbalanced datasets.

#### 4.2.5. Brain regions backtracking

Following the classification process, we selected features or voxels that demonstrated a high level of accuracy. To filter out irrelevant features and pinpoint the most informative voxels that can differentiate differences in functional activations, we utilized methods like GLM. To determine the corresponding brain regions of the chosen voxels, we employed brain backtracking. This entailed transforming the indexes of the selected voxels into Cartesian coordinates, which were then converted into MNI space via the EPI template. We then used the Talairach Daemon toolbox to convert all MNI coordinates into Talairach space coordinates. To visualize and identify the brain regions, we created a mask of the positions of the selected voxels using the MANGO tool, overlaying it onto a normalized template fMRI image. Finally, our proposed approach was applied to each subset of data for all analyzed symptoms, revealing the names of the affected brain areas.

## 5. Results

**Figure 2:**
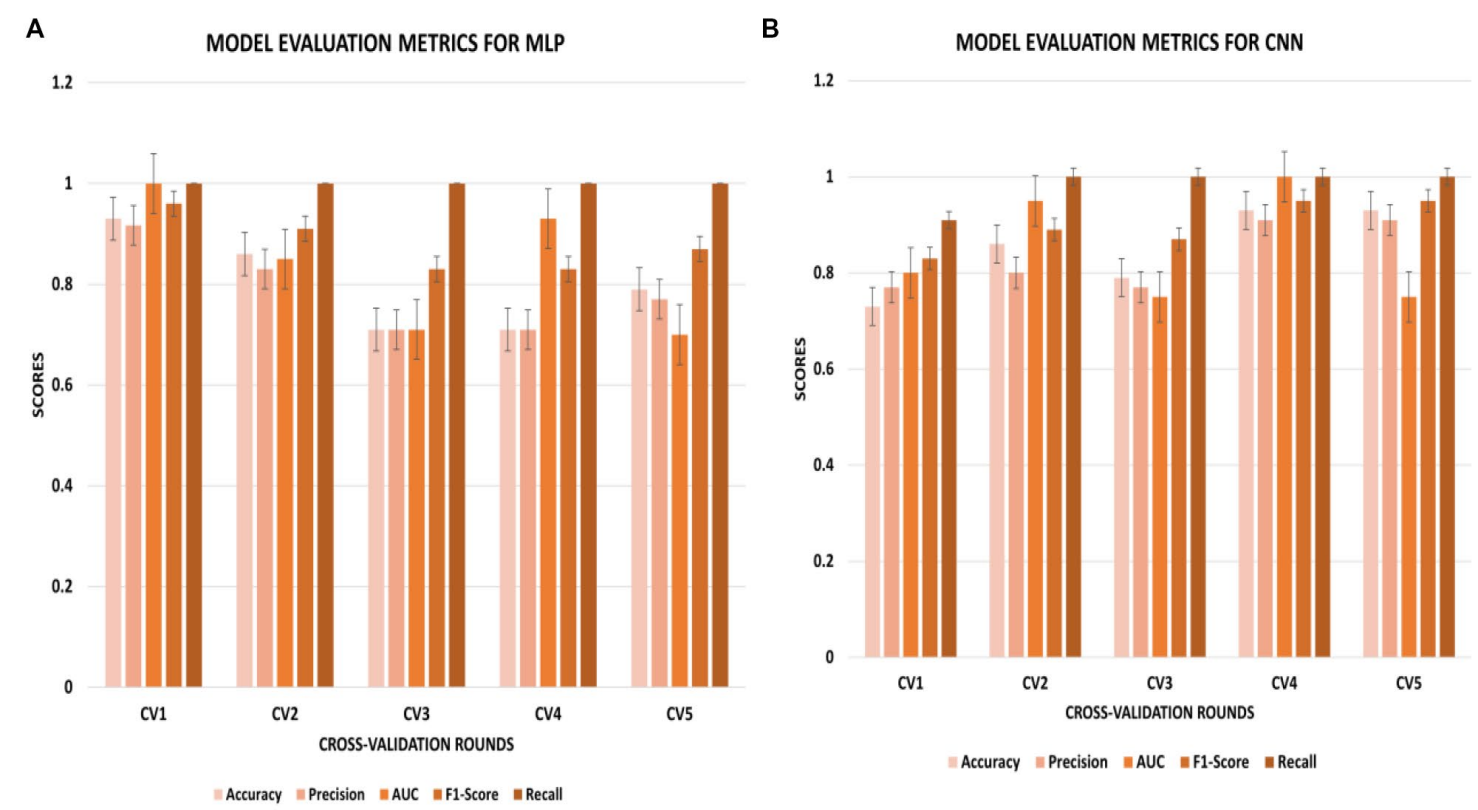
**(A,B)** Metric chart for accuracy, AUC, precision, recall, and F1-score values for **(A)** MLP results; **(B)** CNN results.

**Figure 3:**
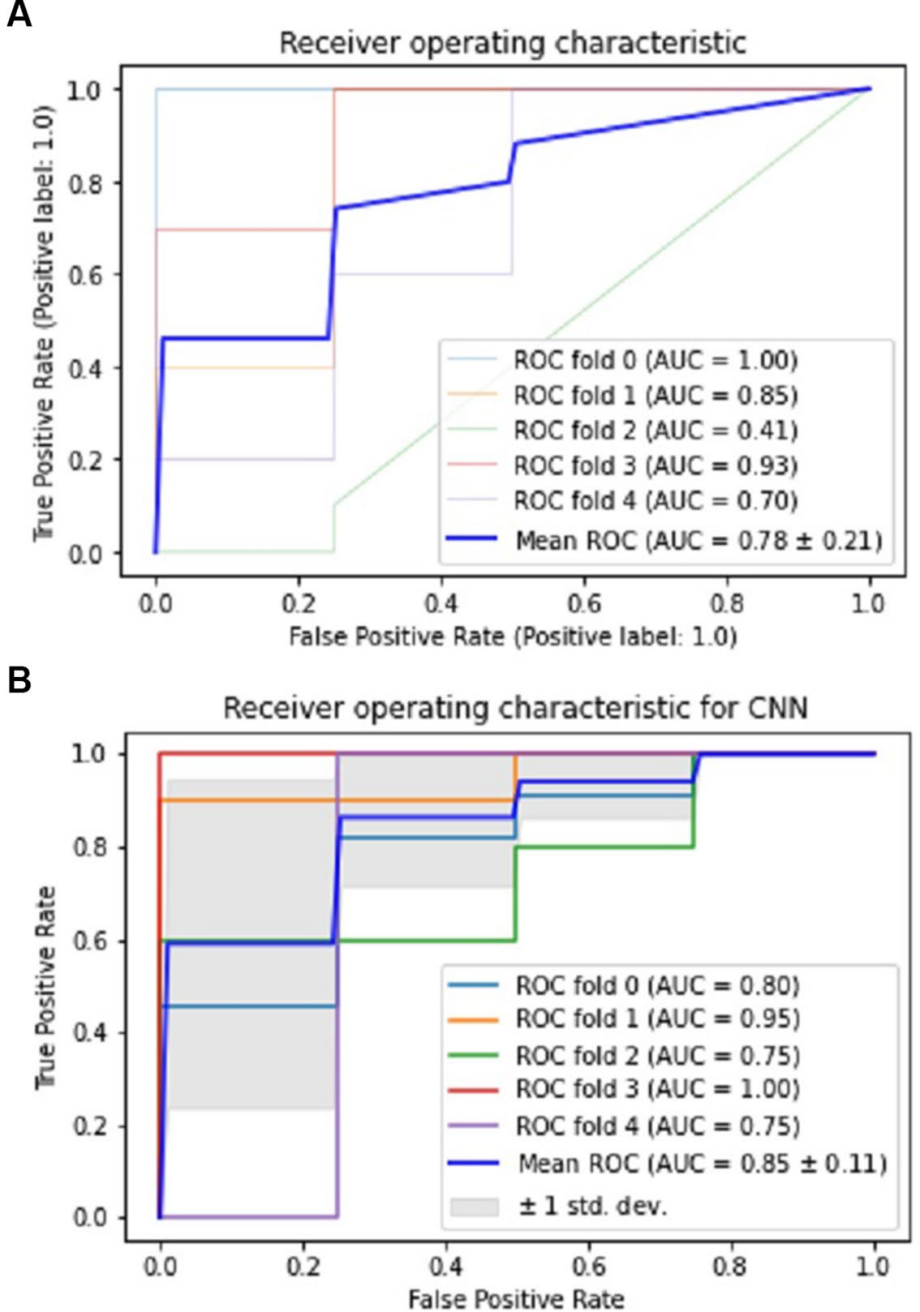
Performance visualization of MLP and CNN using ROC and AUC. **(A)** ROC and AUC performance of MLP. **(B)** ROC and AUC performance of CNN.

The CNN experiments using the configurations described in Section 4 produced the best results, with a mean accuracy of 85.20% and a standard deviation of 0.08. The average AUC was 0.85, as indicated in Table 3. The charts for all the calculated metrics are shown in Figures 2A,B for MLP and CNN, respectively. These line charts depict accuracy, AUC, precision, recall, and F1-Score values for the experiments. The CNN’s performance is illustrated with a ROC curve in Figure 3B.

**Table 3:** Results showing all the metrics of the experiments.

	CNN		MLP	
	Mean	Std Dev (+/−)	Mean	Std Dev (+/−)
Accuracy	85.20%	0.08	80%	0.09
AUC	0.85	0.11	0.78	0.07
Precision	0.83	0.06	0.79	0.08
Recall	0.98	0.04	1	0
F1-Score	0.9	0.05	0.88	0.05

The results obtained from the MLP showed an accuracy of 80% with a standard deviation of 0.09 and an average AUC of 0.78 using the parameters described in Section 4. The performance of the MLP is illustrated in Figure 3A. Table 3 shows an overview of the evaluation parameters, such as precision, recall, and F1-score, for each round of the 5-fold CV. It can be observed that in all 5 rounds of cross-validation with a dataset of 20 healthy controls and 51 pain patients, all 51 pain patients were correctly classified as pain patients, 6 healthy controls were correctly classified as healthy, and 14 healthy controls were misclassified as pain patients. Interestingly, no pain patient was misclassified as a healthy control.

On the other hand, the CNN achieved a mean accuracy of 85.2% with a standard deviation of 0.08 and an average AUC of 0.85, as reported in Table 3. The performance of the CNN is illustrated in Figures 2B, 3B, which show the line charts for accuracy, AUC, precision, recall, and F1-score values. The CNN was trained over 5 cross-validation rounds, and the results showed that 48 pain patients were correctly classified, along with 12 healthy controls. However, 8 healthy controls were misclassified as pain patients, and only 3 pain patients were wrongly classified as healthy controls, indicating a relatively low error rate in comparison to the correctly classified pain patients.

The identified brain regions and their involvement in chronic pain in OA patients can provide important insights into the underlying mechanisms of pain perception and can help develop new treatment strategies. The frontal lobe’s involvement suggests that pain processing and perception involve higher cognitive functions, such as attention and decision-making. The temporal lobe’s involvement suggests that pain perception involves the processing of sensory information, such as auditory and visual stimuli. The parietal lobe’s involvement suggests that pain perception involves the integration of sensory information from multiple modalities. The limbic lobe’s involvement suggests that pain perception is related to emotional and motivational processes.

The identified affected areas on the gyrus level, such as the middle frontal gyrus and superior temporal gyrus, are consistent with previous studies on chronic pain. These regions are involved in the processing of pain-related cognitive and emotional aspects. The involvement of the precuneus gyrus and precentral gyrus suggests that pain perception involves self-referential and motor-related processes. The involvement of the cingulate gyrus and insula is consistent with their known roles in pain processing and modulation.

**Figure 4:**
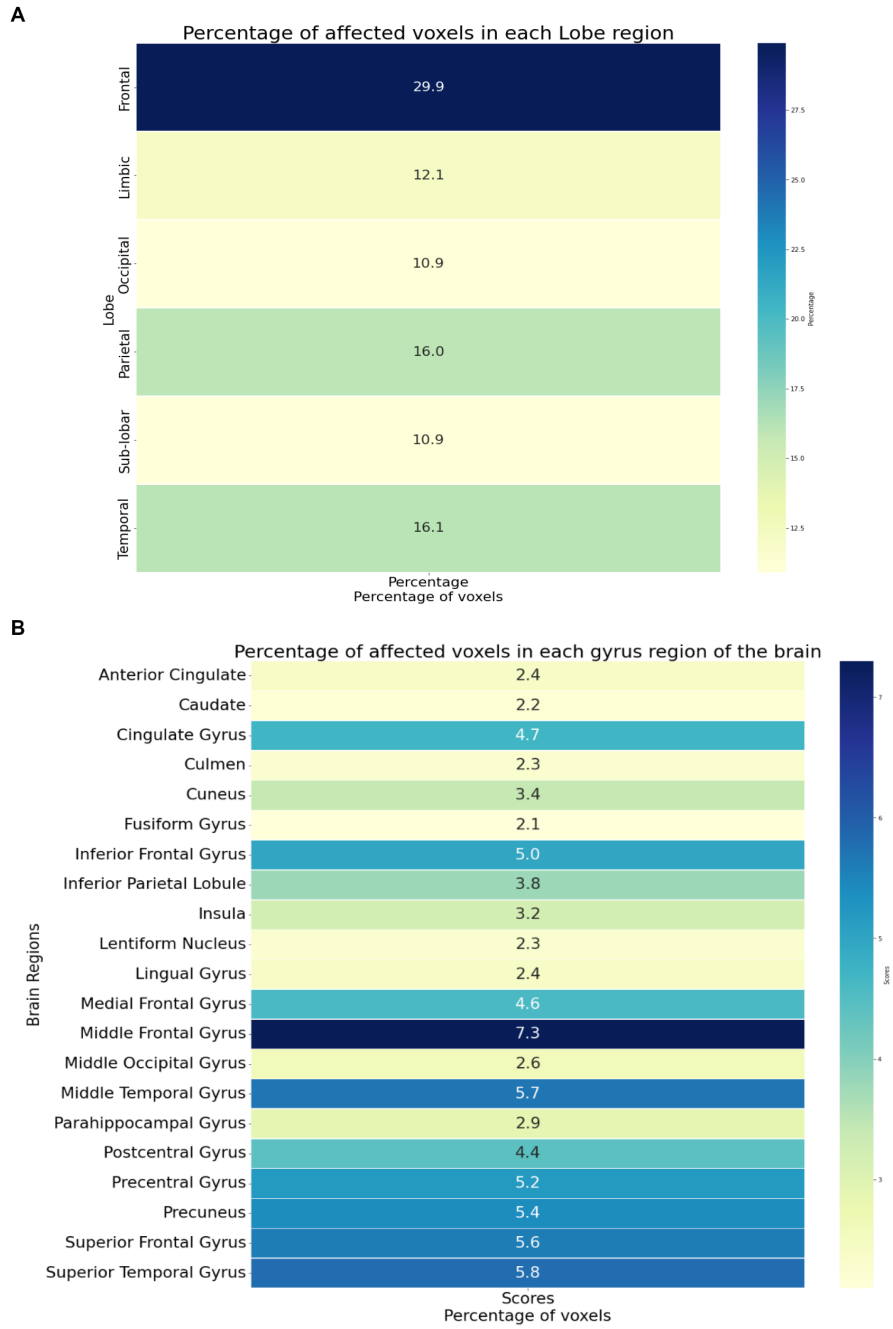
**(A)** The brain regions showing the significant brain activities on the lobe level. **(B)** The brain regions showing the significant brain activities on the gyrus level.

**Figure 5:**
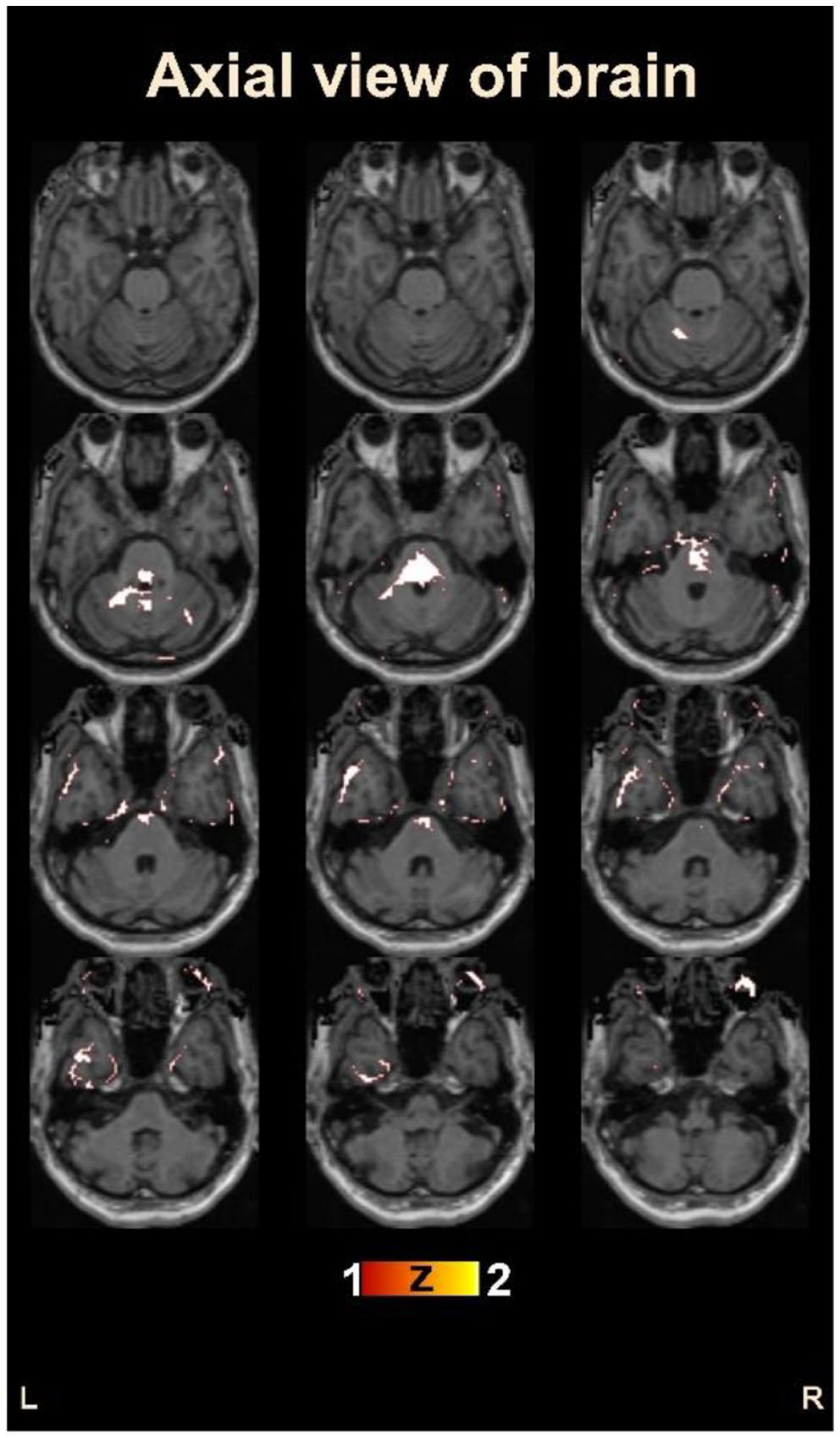
Visualization of the identified brain areas potentially responsible for chronic pain in osteoarthritis patients.

Figure 4 provides an overview of the brain areas affected in terms of lobe and gyrus levels. The identified features were initially traced back to the Cartesian coordinate, followed by MNI space, and finally to the Talairach space. They were then overlaid onto a functional brain image. The visualization of brain regions potentially responsible for pain sensation in chronic pain in OA patients is shown in Figure 5. These results provide valuable insights into the neurological basis of chronic pain and could potentially lead to more effective treatments for OA patients.

## 6. Discussion

Our investigation revealed that no previous research has employed CNN to study rs-fMRI data of OA patients experiencing chronic pain. As such, this study sought to explore the potential of DL algorithms in this field, utilizing both MLP and CNN architectures without any feature selection methods.

Importantly, neither of these DL architectures employed pre-trained weights or transfer learning, and as such, achieved their best performance based on training exclusively on this fMRI dataset. Of these two approaches, the proposed CNN architecture achieved the most promising results, demonstrating a classification accuracy of 85.2% and an AUC value of 0.85. Our findings also showed that the CNN outperformed the MLP in terms of classification accuracy and was better able to identify healthy controls. Furthermore, this high accuracy was achieved without reducing the total number of voxels in the dataset, which adds to the significance of our findings.

As the first study of its kind to employ a CNN-based model for automated diagnosis of OA patients, no comparable research exists for direct comparison. However, we did identify a few CNN-based fMRI studies in other illness domains, where our study’s classification accuracy outperformed previous works (18, 19). This achievement demonstrates the efficacy of our study and contributes to the field with a DL-based computer-aided tool for classifying healthy controls and chronic pain patients.

Furthermore, our study was able to identify the brain regions responsible for chronic pain in OA patients. We found that a few brain regions identified in this study, such as the frontal lobe (29, 39) and the parietal lobe (29, 39), have been previously associated with OA patients at the lobe level. Our findings also identified additional brain regions that have been previously linked to OA patients at the gyrus level, such as the middle frontal gyrus (40, 41) and the superior temporal gyrus (41), thus strengthening the efficacy of our study. Additionally, our study identified the cingulate gyrus, a region that has been previously investigated in OA pain patients (41) but has also been shown to be involved in general pain sensation.

Importantly, our study also identified several brain regions that have not been previously associated with OA pain, such as the superior frontal gyrus, cuneus, middle occipital gyrus, and culmen. These novel findings have the potential to lead to improved treatments and clinical interventions for chronic pain patients in the future. Our study’s novel insights suggest that these brain regions may be potentially responsible for pain sensation in chronic OA patients, and additional research in this direction may lead to new therapeutic targets and approaches.

The findings of our study have important implications for the diagnosis and treatment of chronic pain in OA patients. In addition to developing a DL-based classification model, our study has also identified new brain regions that were never identified in previous studies. These regions, including the superior frontal gyrus, cuneus, middle occipital gyrus, and culmen, are potentially responsible for pain sensation in chronic OA patients.

Chronic pain and OA have been associated with structural and functional changes in the brain. Neuroimaging studies have shown alterations in brain regions involved in pain processing, such as the anterior cingulate cortex, insula, thalamus, and prefrontal cortex (42, 43). In addition, studies have suggested that the brain’s default mode network, which is involved in self-referential processing and mind-wandering, is also involved in chronic pain (44).

In the context of the brain regions identified in our study, the occipital lobe is involved in visual processing and has been implicated in the perception of pain-related visual cues (45). The superior frontal gyrus, located in the frontal lobe, is known to play a role in the modulation of pain perception and the processing of emotional and cognitive aspects of pain. It has been associated with cognitive control and decision-making processes, which may be involved in pain coping strategies (46). The cuneus, located in the occipital lobe, is involved in visual processing and has been linked to the processing of pain-related stimuli. The middle occipital gyrus, also located in the occipital lobe, is involved in visual perception and has been implicated in the processing of painful stimuli. The culmen, located in the cerebellum, is involved in sensorimotor integration and proprioception and has been linked to the perception of pain intensity. It has also been implicated in the modulation of pain perception and the processing of pain-related emotions (47).

Understanding the functional changes that occur in the brain in response to chronic pain and OA is crucial in shedding light on the underlying mechanisms of these conditions. Consequently, it is important to explore the specific brain regions involved in pain processing as this knowledge can aid in the development of targeted treatments to improve pain management and the quality of life for individuals living with these conditions (48).

Furthermore, the identification of novel brain regions in this study adds to the existing literature and provides a more comprehensive understanding of the neurological basis of chronic pain in OA patients. This expands the current knowledge in the field and provides a foundation for future research to investigate the neural mechanisms underlying chronic pain in this patient population. This study’s results, which identify multiple brain regions involved in chronic pain in OA patients, are of significant clinical importance. The outcomes of this study could lead to the development of new and innovative treatment approaches and interventions for chronic pain management. Such advances in the field are critical in improving patient outcomes and quality of life.

Our study primarily focused on chronic pain in patients with OA; however, the DL framework we developed has the potential to be applied to other chronic pain conditions. The brain regions identified in our study, including the occipital lobe, superior frontal gyrus, cuneus, middle occipital gyrus, and culmen, have been previously implicated in chronic pain conditions beyond OA (49, 50). For example, studies have shown altered activity in the anterior cingulate cortex and insula, two brain regions involved in pain processing that were also identified in our study, in patients with fibromyalgia and neuropathic pain (51, 52). Moreover, the DL framework we developed has the potential to improve the accuracy of pain diagnosis and the development of targeted pain therapies for a variety of chronic pain conditions (53). Therefore, we strongly believe that our study has important implications beyond OA and can contribute to a deeper understanding of the neurobiological basis of chronic pain.

Our findings shed light on the neural mechanisms underlying chronic pain in OA patients. However, we acknowledge certain limitations. The small sample size reduces the statistical power of our findings. Furthermore, our sample contains a higher proportion of OA patients than healthy controls. To address the problem of unbalanced sample size, we used powerful statistical techniques that can handle imbalanced data. Welch’s *t*-test, which can accommodate different variances and sample sizes, was used. Additionally, permutation tests were used, which even with unbalanced samples yield accurate *p*-values. These techniques allowed us to obtain valid statistical results and derive meaningful conclusions from our analysis. Despite these limitations, our findings highlight the promise of ML and DL approaches for better understanding the neural correlates of chronic pain in OA patients. To validate our findings, we encourage future researchers to use larger and more balanced datasets.

Finally, utilizing DL-based approaches in this study highlights the potential of this methodology to advance our understanding of chronic pain and develop more effective treatment approaches. Therefore, this study underscores the importance of further research in this area, as it may help reduce the stigma associated with chronic pain and provide a better understanding of the biological basis of this condition. Overall, this study’s findings contribute to the growing body of literature on chronic pain and provide a valuable foundation for future research in this field.

## 7. Conclusion

Our study provides novel insight into the ability of DL algorithms in analyzing rs-data of OA patients experiencing chronic pain. Our proposed CNN architecture achieved the most promising results, marking a significant milestone in the field of chronic pain research. It is worth noting that our study achieved high accuracy without reducing the total number of voxels in the dataset, indicating the robustness of our findings. Our research also identified several brain regions, including the superior frontal gyrus, cuneus, middle occipital gyrus, and culmen, that were not previously associated with OA pain. These novel findings provide a deeper understanding of the neurobiological basis of chronic pain in OA patients and open up new avenues for further research. By identifying these previously unknown brain regions, our study has the potential to aid in the development of targeted and effective pain therapies and interventions, ultimately improving the quality of life for chronic pain patients. Incorporating neuroimaging techniques, in conjunction with our CNN architecture, into clinical practice could be a game-changer in the field of pain management.

## Data availability statement

Publicly available datasets were analyzed in this study. This data can be found here: openfmri.org (accession number: ds000208).

## Ethics statement

Ethical review and approval was not required for the study on human participants in accordance with the local legislation and institutional requirements. Written informed consent from the patients or patients legal guardian/next of kin was not required to participate in this study in accordance with the national legislation and the institutional requirements.

## Author contributions

IC identified the problem statement and hypothesized the solution. IC and LB performed the experiments and wrote the first draft. IC and MC performed the manuscript’s final writing and result analysis. All authors contributed and performed the study.

## Funding

This work was supported by a National Research Foundation of Korea (NRF) grant funded by the Korean government (MSIT) (No. 2021R1A2B5B01001789).

## Conflict of interest

The authors declare that the research was conducted in the absence of any commercial or financial relationships that could be construed as a potential conflict of interest.

## Publisher’s note

All claims expressed in this article are solely those of the authors and do not necessarily represent those of their affiliated organizations, or those of the publisher, the editors and the reviewers. Any product that may be evaluated in this article, or claim that may be made by its manufacturer, is not guaranteed or endorsed by the publisher.
